# Phenylketonuria screening in the Republic of Macedonia

**DOI:** 10.1186/s13023-016-0483-2

**Published:** 2016-08-05

**Authors:** Mirjana Kocova, Violeta Anastasovska

**Affiliations:** 1 Department of Endocrinology and Genetics, University Children’s Hospital, Vodnjanska 17, Skopje, 1000 Republic of Macedonia; 2 Laboratory for neonatal screening, University Children’s Hospital, Vodnjanska 17, Skopje, 1000 Republic of Macedonia

**Keywords:** Phenylketonuria, Neonatal screening, Tandem mass spectrometry

## Abstract

Phenylketonuria is an autosomal recessive inborn error of metabolism which can be prevented by early and continuous treatment. Therefore newborn screening for phenylketonuria has been introduced in many countries. We present here the results of the selective newborn screening for inborn errors of metabolism, including PKU, performed by tandem mass spectrometry which has been introduced in Macedonia since 2011.

## Letter to the editor

Phenylketonuria (PKU) is an autosomal recessive inborn error of metabolism (OMIM 261600) which impairs postnatal cognitive development, a consequence that can be prevented by early and continuous treatment with a semi-synthetic low-phenylalanine diet. PKU was among the first of the human genetic diseases to be recognised as potentially treatable [1], therefore PKU newborn screening has been introduced in many countries [2]. The overall prevalence of PKU phenotypes in European populations approximates 1/10,000 births [3].

Tansek Z et al. recently reported in Orphanet Journal of Rare Disease assessment of the current state of PKU screening and management in the region of southeastern Europe [4]. The survey included 11 countries from South-Eastern region of Europe including Macedonia. This report contains incorrect data for the PKU screening program and GDP per capita in Macedonia. The authors claimed that PKU newborn screening was not introduced in 4 out of 11 countries: Albania, Kosovo, Macedonia and Montenegro. However, in Macedonia selective newborn screening for inborn errors of metabolism, including PKU, as a part of the National program for mothers and children’s care of the Ministry of Health of Macedonia, has been introduced since 2011. It is performed by tandem mass spectrometry (LC/MS/MS—Liquid chromatography—tandem mass spectrometry). Six larger nurseries from all regions of the country were covered by the selective screening for metabolic disorders. The newborn screening bloodspot specimens were collected 48–72 h after birth. During 2012, 4072 newborns were screened and one newborn with phenylketonuria was detected and diagnosed subsequently. Amino acid analysis of the patient with PKU showed markedly elevated phenylalanine, 1802 μmol/L (reference range 0–150) and tyrosine value on the lower border, 26 μmol/L (0–350). The phe/tyr ratio, 69.3, was significantly elevated. Newborn screening for metabolic diseases in Macedonia includes quality control by CDC (Centers for Disease Control and Prevention, http://www.cdc.gov/ncezid/pdf/2015-2017_gap_clia_certificate.pdf) Atlanta, USA.

The interpretation of the economic backgrounds of the participating countries showed that four out of 11 countries had GDP per capita under 10,000 USD (Albania, Bosnia and Herzegovina, Kosovo, Moldova). Macedonia, with GDP 11,834 USD per capita was included in the countries with GDP under 20,000 USD. These data correspond to GDP in International dollars (Int$) and are significantly higher than GDP in USD. All discussion in the paper is about GDP in USD, however, given GDP values are in International dollars. Report of the State Statistical Office of the Republic of Macedonia for Gross domestic product, 2001–2014 [5], showed that GDP per capita for 2012 was 3680 EUR (4127 USD). Thus, Macedonia belongs to the group of countries with low GDP.

In conclusion, in Macedonia, although a country with the low GDP per capita selective neonatal PKU screening is introduced, as a part of newborn screening for more than 30 inherited metabolic disorders, by tandem mass spectrometry in a single test. Activities to cover all newborns are underway.
